# Highly Specific Gene Silencing by Artificial miRNAs in Rice

**DOI:** 10.1371/journal.pone.0001829

**Published:** 2008-03-19

**Authors:** Norman Warthmann, Hao Chen, Stephan Ossowski, Detlef Weigel, Philippe Hervé

**Affiliations:** 1 Department of Molecular Biology, Max Planck Institute for Developmental Biology, Tübingen, Germany; 2 International Rice Research Institute (IRRI), Metro Manila, Philippines; University of Chicago, United States of America

## Abstract

**Background:**

Endogenous microRNAs (miRNAs) are potent negative regulators of gene expression in plants and animals. Artificial miRNAs (amiRNAs)–designed to target one or several genes of interest–provide a new and highly specific approach for effective post-transcriptional gene silencing (PTGS) in plants.

**Methodology:**

We devised an amiRNA-based strategy for both japonica and indica type strains of cultivated rice, *Oryza sativa*. Using an endogenous rice miRNA precursor and customized 21mers, we designed amiRNA constructs targeting three different genes (*Pds*, *Spl11,* and *Eui1/CYP714D1*). Upon constitutive expression of these amiRNAs in the varieties Nipponbare (japonica) and IR64 (indica), the targeted genes are down-regulated by amiRNA-guided cleavage of the transcripts, resulting in the expected mutant phenotypes. The effects are highly specific to the target gene, the transgenes are stably inherited and they remain effective in the progeny.

**Conclusion/Significance:**

Our results not only show that amiRNAs can efficiently trigger gene silencing in a monocot crop, but also that amiRNAs can effectively modulate agronomically important traits in varieties used in modern breeding programs. We provide all software tools and a protocol for the design of rice amiRNA constructs, which can be easily adapted to other crops. The approach is suited for candidate gene validation, comparative functional genomics between different varieties, and for improvement of agronomic performance and nutritional value.

## Introduction

Analyzing naturally occurring loss-of-function alleles and screening large collections of chemically induced or sequence-indexed insertion mutants have proven instrumental to discover gene functions and biological mechanisms. However, saturating entire genomes by this approach requires very large populations and it is restricted to specific genetic backgrounds (varieties). A current challenge in rice research is still to unravel the functions of all genes in the genome, which would ultimately facilitate the identification of genes for agronomically important traits and the linkage between gene functions and specific traits across different varieties. Perhaps contrary to intuition, there are several examples where loss of gene function results in improved plant performance, such as increased yield or biotic and abiotic stress tolerance [1]–[3]. One prominent example in rice is *sd1*, which encodes a GA_20_ oxidase, *GA20ox-2*, involved in gibberellin biosynthesis. Inactivation of *Sd1* results in a semi-dwarf phenotype, a trait that triggered the Green Revolution in rice. Since the 1960s, *sd1* alleles have been widely used to confer semi-dwarfism in modern rice cultivars [4]–[6]. Similarly, loss of activity of *Eui1* (*Elongated uppermost internode1/CYP714D1*), which encodes a cytochrome P450 monooxygenase, promotes panicle exsertion, a trait useful in male sterile lines for hybrid seed production [7], [8]. Originally identified as a spontaneous mutant in a japonica variety, the original *eui1* allele had to be introduced into indica varieties by introgression or new alleles had to be isolated after mutagenesis in the desired background [9 and references therein].

Today transgene-mediated gene silencing through RNA interference (RNAi) offers a directed way of inactivating one or several specific genes [10]–[12]. RNAi transgenes are dominant and can be applied in many different genetic backgrounds for any known gene in the genome. In addition it enables, for example, simultaneous targeting of several sequence-related genes, including duplicate genes in polyploids or redundantly acting homologs. Pathogen-derived genes have also successfully been targeted, e.g., to achieve enhanced virus and insect resistance [2], [13]–[15]. Through tissue-specific, inducible or stimuli-responsive and partial gene inactivation, desired mutant phenotypes may be temporally and/or spatially restricted and modulated in severity. Although widely used for gene discovery and validation of gene function, one impediment to a broader use of transgene-mediated gene silencing especially in crop improvement has been the question of specificity.

In the 1990s, several related transgenic approaches to induce loss of gene function in plants were developed. They were later found to all act through small silencing RNAs (sRNAs) derived from double-stranded RNA precursors [16], [17]. Surprisingly, only few studies have systematically compared different silencing strategies. Two of these found that hairpin RNA interference (hpRNAi) produced more efficient silencing triggers than separately transcribed sense and antisense RNAs [18], [19]. Although most efforts to improve sRNA-mediated gene silencing have focused on maximal effectiveness, a recent computational analysis from 25 plant species predicted that a majority of transcribed regions have potential off-target effects when used in post-transcriptional gene silencing (PTGS) [20]. Potential off-target effects of hpRNAi in plants arise because the large number of sRNAs spawned by the silencing construct may target any gene that shares perfect or near-perfect complementarity to these sRNAs. Long hairpins are more likely to give rise to optimally effective sRNAs, but the chance of generating sRNAs with unwanted off-target effects increases as well.

The recently developed artificial microRNA (amiRNA) technology exploits endogenous miRNA precursors to preferentially generate a single specific sRNA *in vivo* [2], [11], [12], [21]–[25]. MiRNAs in plants typically induce cleavage of the target mRNA opposite positions 10 and 11 of the miRNA [26]. Because the amiRNA sequence does not have to be perfectly complementary to the target site, it can be optimized to target only one or, alternatively, several sequence-related genes. Genome-wide expression analyses have shown that amiRNAs have similarly high specificity as endogenous miRNAs [21], [27]. Efficient amiRNA-mediated gene silencing has been observed to occur in a quantitative fashion, with stronger promoters often causing higher degrees of gene silencing and it seems that there are few, if any non-autonomous effects [21], [22], [25]. *Arabidopsis* miRNA precursors have been modified to silence endogenous and exogenous target genes in the dicotyledonous plants *Arabidopsis*, tomato and tobacco [2], [13], [21]–[23], and because of their specificity and versatility, amiRNA vectors are recognized as second-generation RNAi vectors [12].

Gene silencing by amiRNAs has not yet been demonstrated for a monocotyledon species, or for traits proven to be of agronomic importance. Here, we report the successful application of amiRNAs to agronomically relevant strains of both japonica and indica rice, together with enabling tools for the breeding and functional genomics communities.

## Results

**Figure 1:**
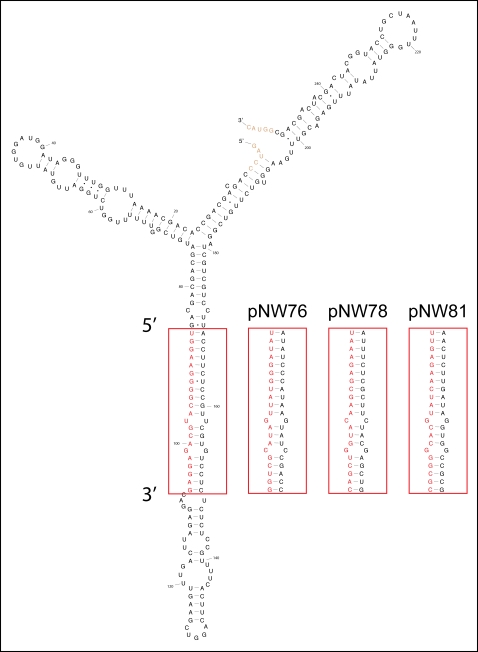
Secondary Structure of the osa-MIR528 stemloop in pNW55. Depicted is the secondary structure of the 255 bp derived from osa-MIR528 (245 bp plus the BamHI and KpNI restriction sites) as predicted by ‘RNAfold’ [33] (Vienna RNA package, http://www.tbi.univie.ac.at/RNA) for 23°C. The sequences replaced in the different transgenes are boxed. The oligo designer in WMD2 [25] (http://wmd2.weigelworld.org) designs the amRNA* sequence (black) such that it pairs to the respective miRNA (red) in the same way as in osa-MIR528.

**Figure 2:**
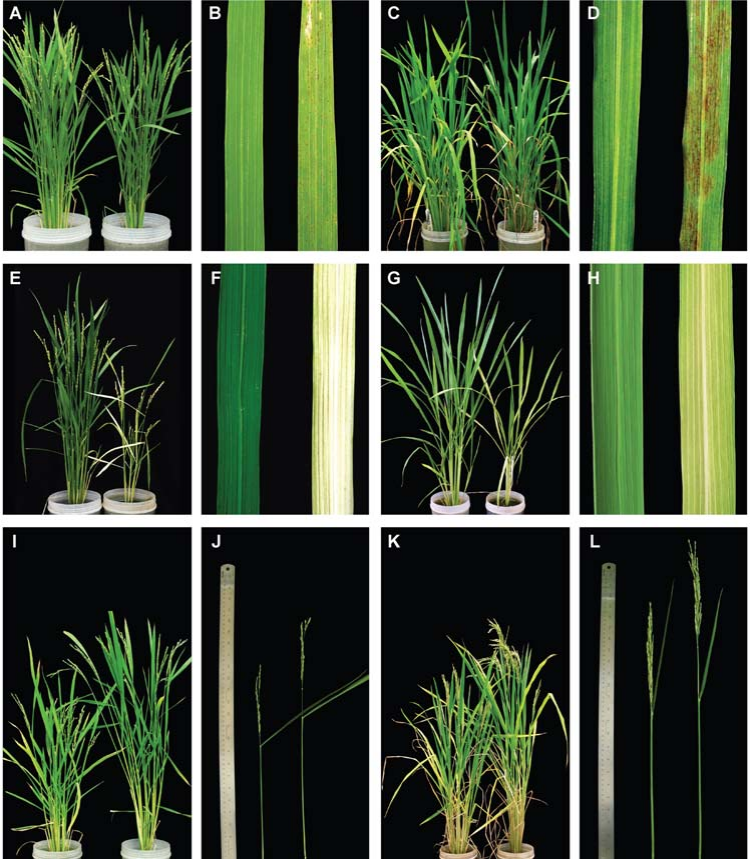
Phenotypes of plants overexpressing amiRNAs. The empty vector control is always on the left. (A–D) pNW76, construct targeting *Spl11*. (A) Nipponbare whole plants, (B) Nipponbare leaves, (C) IR64 whole plants, (D) IR64 leaves at maximum tillering stage. (E–H) pNW78, construct targeting *Pds*. (E) Nipponbare whole plants, (F) Nipponbare leaves, (G) IR64 whole plants, (H) IR64 leaves at tillering stage. (I–L) pNW81, construct targeting *Eui1*. (I) Nipponbare whole plants, (J) Nipponbare uppermost internode, (K) IR64 whole plants, (L) IR64 uppermost internode. The ruler in (J) and (L) is 60 cm long.

To optimize expression of the amiRNA, we chose to employ an endogenous rice miRNA precursor, a 245 bp fragment of the osa-MIR528 locus. Appropriate 21mers to replace the endogenous miRNA and miRNA* of osa-MIR528 were designed based on the TIGR4 rice genome annotation. Modified precursors (**Figure 1**) were constitutively expressed in two rice varieties, Nipponbare (japonica) and IR64 (indica). The method was evaluated by targeting three different rice genes, *Phytoene desaturase* (*Pds*, Os03g08570), *Spotted leaf 11* (*Spl11*, Os12g38210), and *Eui1* (*CYP714D1*, Os05g40384) (**Table 1**). Mutations in these genes cause an albino phenotype (*pds*) [28], spontaneous lesion formation in the absence of pathogens (*spl11*) [29], and elongation of the uppermost internode at heading stage (*eui1*) [7], [8], respectively. Transgenic lines containing the amiRNA constructs reproduced the previously described phenotypes for all intended target genes in both varieties (**Figure 2** **,** **Table 2** **,** **Table S1**).

**Table 1:** Target genes and miRNA sequences.

Target gene	Locus	Loss-of-function phenotype	amiRNA clone	Predicted mature miRNA sequences
*Spl11*	Os12g38210	Spotted leaves	pNW75	TAAGGCGAGTGATTCATGCGT
			pNW76	TATAGGGTATTGATACGCTGG
*Pds*	Os03g08570	Albino	pNW77	TTAAGAATTACTATGCAGGCC
			pNW78	TAAAGAGCGAACATGGTCGAC
*Eui1*	Os05g40384	Elongated upper-most internode	pNW81	TTGAGAACTATGCACGGGCGC
			pNW82	TAGTTCACGACTTACTAGGTG

**Table 2:** Efficiency of different amiRNA constructs in Nipponbare and IR64 T_0_ plants.

amiRNA vector	Target gene	Nipponbare			IR64		
		No. of independent transformants	Mutant mimics	Efficiency (%)	No. of independent transformants	Mutant mimics	Efficiency (%)
**pNW75**	*Spl11*	33	0	0	7	0	0
**pNW76**	*Spl11*	29	15	51.7	32	12	37.5
**pNW77**	*Pds*	38	0	0	21	0	0
**pNW78**	*Pds*	42	39	92.9	68	38	55.9
**pNW81**	*Eui1*	32	9	28.1	40	33	82.5
**pNW82**	*Eui1*	35	0	0	8	0	0

Phytoene desaturase (PDS) catalyzes an early step in the carotenoid biosynthesis pathway and the absence of protective carotenoids results in bleaching through photo-oxidation of chlorophyll, making it a convenient gene for proof-of-principle applications. PDS is encoded by a single copy gene in the rice genome and silencing of *Pds* (AF049356) in rice by hpRNAi, using a 470 bp gene fragment, has been reported to cause an albino phenotype [28]. Over 90% (39 of 42) of transgenic Nipponbare plants (NB_pNW78) and 56% (38 of 68) of transgenic IR64 plants (IR64_pNW78) had an albino phenotype (**Figure 2** **,** **Table 2**). Many plants with severe bleaching died soon after transfer to rooting medium or soil (**Figure S1**).

The original rice *spl11* mutant had been identified from an EMS mutagenized IR68 (indica) population and shows rust colored spots from maximum tillering stage to maturity [30]. Fifty-two% (Nipponbare, *n* = 29) and 38% (IR64, *n* = 32) of independent transgenic lines showed the characteristic phenotype (**Figure 2** **,** **Table 2**). Both sets of transgenic plants, NB_pNW76 and IR64_pNW76, exhibited leaf lesions at tillering stage, with the mutant phenotype being most severe at the maximum tillering stage. Although the observed efficiency based on the phenotypic data seems low for IR64, we detected reduced expression of *Spl11* transcript in 18 out of 20, or 90%, of primary IR64_pNW76 transformants, (data not shown). We subsequently compared T_1_ progeny of four independent IR64_pNW76 lines to three IR64 *spl11* mutants (G5612-1-6, G5717-1-6, and D2487-3-5). In the mutants as well as in the pNW76 T_1_ plants we observed the characteristic leaf spots already at the 4-leaf stage, even though we did not detect the mutant phenotype at this early stage in the T_0_ lines. We attribute the weak phenotype in T_0_ plants to environmental conditions and to the effects of in-vitro regeneration (Guo-Liang Wang, personal communication). These data suggest that the silencing efficiency based on the phenotype of T_0_ lines reported in **Table 2** may be an underestimate.

*Eui1* encodes a cytochrome P450 monooxygenase (CYP714D1) that inactivates gibberellin (GA) and thus modulates GA-dependent development [8]. It is preferentially expressed in young panicles during heading stage and loss of EUI1 activity results in increased availability of biologically active gibberellin in the uppermost internode [8]. Plants carrying *eui1* mutations are taller than wild-type plants owing to increased internode lengths, most extreme in the uppermost internode [7], [8]. Transgenic plants expressing an amiRNA construct against *Eui1* (pNW81) were taller than control plants with significantly longer uppermost internodes and better panicle exsertion (**Figure 2** **,** **Table S1**). 83% (33 of 40 independent transformants) of IR64_pNW81 plants and 28% (9 of 32) NB_pNW81 plants exhibited the characteristic *eui1* phenotype (**Table 2**). The phenotype was more pronounced in the indica variety IR64, which is in agreement with reports on different *eui1* alleles in indica and japonica varieties [7]. Although the phenotypic data indicate a low efficiency for Nipponbare, we found the expression of *Eui1* transcript to be clearly reduced in 15 out of 19 (79%) of Nipponbare_pNW81 plants (data not shown). This again indicates that the actual silencing efficiency might be higher than indicated in **Table 2**, but that the phenotype in T_0_ plants was attenuated by environmental and in-vitro regeneration conditions.

Among the transgenic plants that exhibited the expected phenotypes, we identified T_0_ plants that contained only one or two copies of the amiRNA transgene (**Figure S2**), which confirms the effectiveness of amiRNA-mediated gene silencing. For three Nipponbare T_0_ lines that contained a single copy of pNW78, we analyzed the progeny (T_1_ plants) and observed Mendelian, dominant inheritance of the albino phenotype (3:1). PCR analysis confirmed that the phenotype co-segregated with the transgene (**Table S2** **,** **Figure S3**).

**Figure 3:**
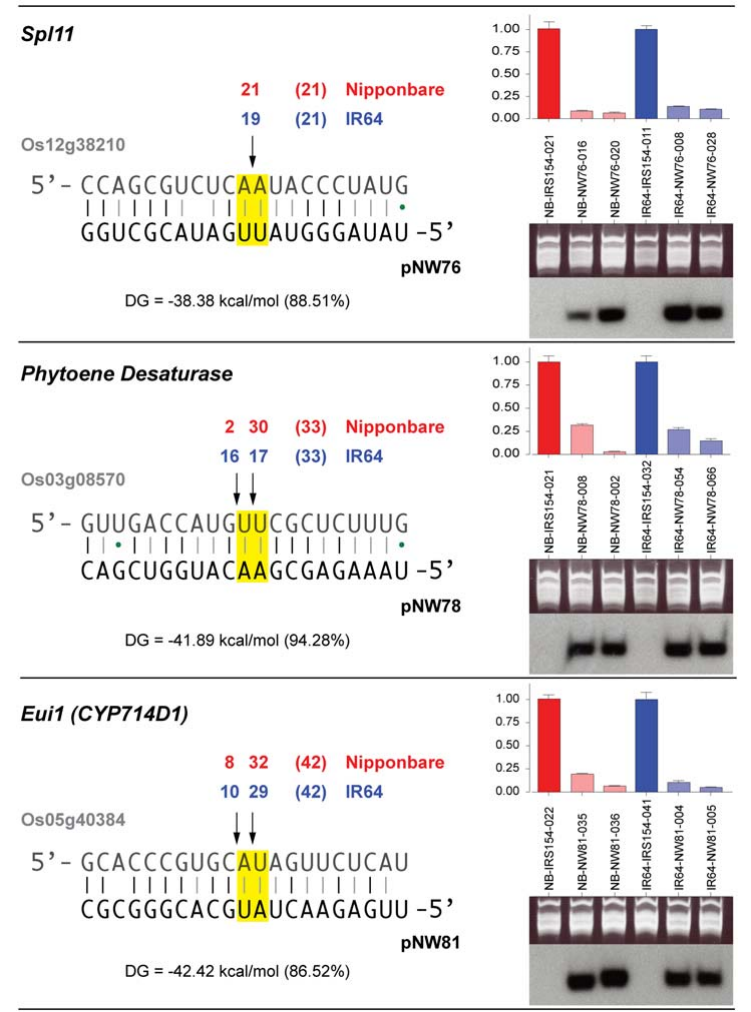
Molecular characterization of transgenic plants. Cleavage site mapping was performed on mRNA from one transgenic plant for each transgene in both varieties (Nipponbare and IR64). Numbers above the arrows denote the number of clones ending at the particular position, with the total number of clones in parentheses. The binding energy (ΔG) of the RNA-RNA duplex between target (denoted by TIGR locus identifier) and amiRNA is given in kcal/mol and as a fraction of the calculated binding energy for a perfect match to the target site. Total RNA from two transgenic plants for each construct (leaf tissue for *SPl11* and *Pds*, young panicles for *Eui1*/*CYP714D1*) was used for RT-PCR for the target (histograms, top right), and small RNA blots (bottom right). Gel images are provided as loading control for small RNA blots. Comparison was to an empty vector control (IRS-154). Expression was normalized to the respective empty vector control. Error bars indicate the variation between technical replicates (range).

To demonstrate that the observed phenotypes are indeed due to amiRNA mediated down-regulation of the target genes, we analyzed the expression levels of the amiRNAs and their target genes in selected transgenic lines for both Nipponbare and IR64. The expected amiRNA species were readily detected by RNA blot in every line and tissue tested, which demonstrates that the amiRNA precursor is efficiently produced and processed *in planta* (**Figure 3**). Using reverse transcription followed by quantitative real time PCR (qRT-PCR), we found the abundance of the target transcripts to be greatly reduced (**Figure 3**). To ensure causality between amiRNA expression and target gene down-regulation, we mapped the amiRNA-guided cleavage sites by RACE-PCR [26]. In all transgenic lines tested, the expected cleavage products were detected (**Figure 3**), which also indicates that the amiRNAs are of predicted sequence.

*Spl11* has several homologs in the rice genome, which might be problematic for conventional gene silencing strategies such as hpRNAi. To examine the predicted specificity of the amiRNA against *Spl11*, we investigated the mRNA levels of *Spl11* and its nine closest homologs (**Table S3**) by multiplex qRT-PCR. We found that only the transcript abundance of the targeted *Spl11* (Os12g38210) was significantly reduced (**Figure S4**). This observation not only confirms the specificity of the amiRNA, but also indicates that production of secondary siRNAs from the primary target gene, which could indirectly silence related genes, should not be a major concern, in line with the observation that so called transitivity effects of miRNAs are rare.

**Figure 4:**
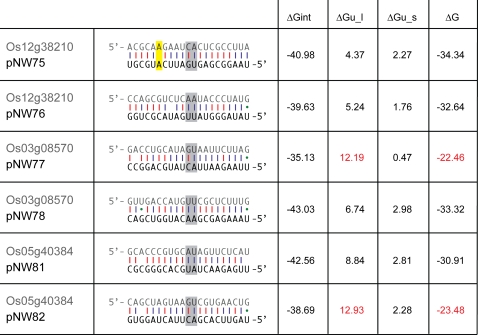
Predicted amiRNA-mRNA interactions. The program ‘RNAup’ [33] was used to assess the thermodynamics of RNA-RNA binding considering 80 bp surrounding the target site at 23°C. The predicted cleavage sites (between positions 10 and 11 of the miRNA) are highlighted in grey. ‘RNAup’ calculates the *effective* free hybridization energy (ΔG) as sum of the energies necessary to open the target site in the target mRNA (ΔGu_l), the internal folding of the amiRNA (ΔGu_s), and the energy gained from hybridization of amiRNA and target mRNA (ΔGint).

We had designed two different amiRNAs for each target gene (**Table 1** **,** **Figure 4**). In each case, one of these was much more effective in both varieties in terms of producing the predicted phenotype (**Table 2**) as well as reducing the RNA levels of the target gene (data not shown). Many aspects of the processing of plant miRNA precursors are still unknown. Some might not be processed as intended, and the resulting amiRNA might therefore not have the predicted sequence. Other possible explanations include sequence properties of the amiRNA or the target site such as target site accessibility that are not optimal. Several recent publications describe important effects of the mRNA structure surrounding the target site on the efficiency of siRNA-mediated gene silencing in animals [31], [32 and references therein]. It has hence been suggested that one should model the thermodynamics of RNA-RNA interactions as the sum of two energy contributions: (1) the energy necessary to ‘open’ the binding site(s) and (2) the energy gained from hybridization [33]. We assessed the thermodynamics of the amiRNA-mRNA interactions for our amiRNAs with their targets using the program ‘RNAup’ of the Vienna RNA package [33]. Two of the three ineffective amiRNAs (pNW77 and pNW82) were found to have unfavorable target sites, as the energy required to ‘open’ the nucleotide bonds at the target sites is high. In contrast, for pNW78 and pNW81, the probability that nucleotides at the respective target sites are unpaired is higher and hence the *effective* hybridization energy of the amiRNA to the target mRNA is better (**Figure 4**). The inefficacy of amiRNA pNW75 is not easily explained by hybridization properties. However, amiRNA pNW75 has a purine:purine mismatch (A:A) to its target mRNA at position 16 (**Figure 4**), which has recently been shown to have a strong negative impact on siRNA mediated silencing in animals [34].

## Discussion

Rice, *Oryza sativa*, is one of the most important staple crops in the world and the model plant for monocots. Still, the majority of rice genes (61% of predicted loci) do not have an assigned or even putative function (Kevin Childs, personal communication). Rice shares excellent gene co-linearity with other monocots and nearly all genes in important crops such as wheat, barley and maize have a homolog in rice [35]. Mutation and activation strategies have been deployed at large scales in rice and other species. Identifying candidate genes for induced mutations or Quantitative Trait Loci (QTL), or establishing parallels between gene functions and specific traits across different species and varieties is difficult and often a rate-limiting step. Directed, specific gene silencing by transgenes provides a powerful tool to tackle these challenges. Our work demonstrates successful gene silencing by amiRNAs in an indica and a japonica variety of rice. Our method is based on a new vector derived from an endogenous rice miRNA precursor, osa-MIR528, and can be easily adopted by the community and expanded to other monocot crops following the experimental procedures described. In our examples, the different mutant phenotypes triggered by amiRNA-directed gene silencing could be rapidly detected in T_0_ lines in different rice varieties, the transgenes were stably inherited and remained active in the progeny. Our data demonstrate that the amiRNA precursor is efficiently processed *in planta* in all tissues tested and the amiRNA-guided cleavage of target transcripts is highly specific.

Although the effects of target site accessibility and the impact of particular mismatches at certain positions on plant miRNA efficiency have not yet been studied systematically, these characteristics may provide a means to deliberately modulate amiRNA efficiency and hence the degree of target gene down-regulation. Our original WMD tool for the design of amiRNAs selected and designed amiRNA sequences based on empirically established criteria [25]. The next generation, WMD2, has been modified such that amiRNA candidates with a purine:purine mismatch to the target mRNA at position 16 are avoided. Based on the results from the current work, it should be possible to improve the design tool by giving more attention to the *effective* binding energy. In any case, designing several amiRNA constructs targeting different regions in the target mRNA remains a prudent avenue. For the analysis of small numbers of genes, the amiRNA constructs are easily produced by modification PCR from the precursor plasmid. In addition, the small size of the miRNA precursor stemloop should facilitate future high throughput production by synthesis.

AmiRNAs will have many applications in rice functional genomics. They can trigger efficient silencing of one or several genes or specific alleles with great specificity, even for genes essentially inaccessible to other reverse genetics techniques, e.g., tandem duplicated genes. Many recent QTL studies have shown that a reduction or loss of gene function often underlies varietal differences and agronomically important traits in rice and other grasses [36]. The amiRNA technology offers a way for time-efficient modification of the expression of such genes in any variety. This not only enables rapid knowledge transfer between different varieties, e.g., between indica and japonica strains, which are separated by complex incompatibility barriers [37], but also the introduction of important traits for improving agronomic performance and/or nutritional value into a broad range of varieties. Its dominant nature and specificity will also be useful in hybrid crop breeding, since only one of the parental lines needs to carry the amiRNA construct and amiRNAs have the potential to specifically reduce the activity of only one of the parental alleles.

## Materials and Methods

### Sequence Information and Software

Rice microRNA precursor sequences were downloaded from miRBase/Rfam (http://microrna.sanger.ac.uk/ [38]), rice gene sequences for WMD from TIGR (http://www.tigr.org/tdb/e2k1/osa1) and 1,140,645 available rice cDNA sequences for blast searches from NCBI (http://www.ncbi.nlm.nih.gov). Sequence homology of miRNA precursors to available rice cDNAs was determined by BLAST and multiple alignments were performed using MUSCLE [39]. The possible amiRNA candidate sequences were generated using WMD [21], [25], customized to use the published *Oryza sativa* genes (TIGR v4, 62,827 sequences) as input. The binding properties of the amiRNAs to the respective target mRNAs (80 bp surrounding the target site) at 23°C (**Figure 4**) were assessed using the program ‘RNAup’ [33]. The top 9 homologs of *Spl11* (Os12g38210) in the rice genome were identified by blasting the *Spl11* CDS against “Genes in TIGR Rice Pseudomolecules-CDS” using WU-BLAST 2.0 implemented on the TIGR Rice Genome Annotation website (http://www.tigr.org/tdb/e2k1/osa1/ [40]. Most of them are annotated as “spotted leaf protein 11, putative” (**Table S3**). Their percent identity to Os12g38210 was determined by pair wise Needleman-Wunsch alignments using the EMBOSS program ‘needle’ with default parameters [41].

### WMD [25]

WMD2 (http://wmd2.weigelworld.org) currently supports 38 plant species including *Oryza sativa* (TIGR v5) and designs 21mer sequences directed against one or several genes. WMD suggests suitable amiRNA candidates after a two-step selection process based on empirically established criteria for efficiency and specificity and is improved as the knowledge on the biology of miRNAs grows [21], [25], [27]. Currently, 21mers from the reverse complement of the target transcript(s) are considered effective amiRNA candidates, if they have an “A” (sometimes also “U”) at position 10 and display 5′ instability (higher AU content at the 5′ end and higher GC content at the 3′ end). The first base (position 1) will be replaced by an “U” and all candidates then undergo an iterative series of in-silico mutations at positions 13–15 and 17–21 followed by mappings against all currently known cDNA sequences or gene models for the particular species. Any transcript other than the specified target(s), which matches the 21mer with up to 5 mismatches, is considered an off-target, if no more than 1 mismatch in the ‘seed-region’ (positions 2–12) is found. Candidates with one or two mismatches to the intended target(s) and without off-targets are then ranked based on hybridization properties and returned to the user.

### The Stemloop Backbone

To select an endogenous stemloop backbone for the amiRNA transgenes we screened all rice miRNA precursors for *Oryza sativa* present in miRBase/Rfam [38] for several criteria: the predicted secondary structure should form a short and simple hairpin-loop and the miRNA* sequence should pair to the miRNA without insertions or deletions. The precursor sequence should be present in EST databases and it should possibly have further proof of its existence and processing in the literature. We selected the precursor of osa-MIR528 (accession number: MI0003201), because of its short stemloop with a predicted simple fold-back structure and its effective expression and processing in several rice tissues including leaves, root and panicle [42]. In addition, MPSS signatures had been detected for the miRNA as well as for the miRNA* in stem tissue of Nipponbare (http://mpss.udel.edu/rice/) [43]. Database searches revealed 3 EST sequences of this precursor, accession numbers: AK099390, AK073820, AK063857. By sequence alignment we noticed that the precursor likely contains an intron. The stemloop of osa-MIR528 is identical in sequence to the annotated rice gene LOC_Os03g03724 (TIGR v5, or LOC_Os03g0129400, RAP). A 245 bp DNA fragment including the osa-MIR528 stemloop, but avoiding the predicted intron, was amplified from Nipponbare genomic DNA by PCR (primers G-11491 and G-11494, **Table S4**, introducing BamHI and KpnI restriction sites), and blunt end cloned into pBluescript KS to give rise to the backbone vector pNW55. All subsequent clones were produced by modification PCR amplifications from pNW55.

### AmiRNA Constructs

We used a customized version of the original Web MicroRNA Designer platform (WMD) to design amiRNA sequences (21mers) based on the TIGR v4 rice genome annotation. WMD suggested suitable amiRNA candidates based on good hybridization properties to the target mRNAs while minimizing possible off-target effects to other genes in the rice genome [25]. We selected two 21mers (amiRNAs) per target gene (**Table 1** **,** **Figure 4**) to target different sites in the target mRNA and designed appropriate amiRNA* sequences that would in pairing to the amiRNA exactly mimic the foldback structure of the endogenous osa-MIR528 (**Figure 1**). Each primary amiRNA construct was engineered from pNW55, replacing the 21 bases of the natural osa-MIR528 miRNA as well as the partially complementary region of the miRNA* by modification PCRs in a similar way as described earlier [21], following the PCR scheme in **Figure S5**. All PCRs were performed with ProofStart ™ DNA Polymerase (Qiagen) in a volume of 25 µl according to the manufacturers recommendation and the PCR protocols are given in **Table S5** **.** For each amiRNA construct, three fragments including the multiple cloning sites (MCS) were PCR amplified from the template clone pNW55 using a total of six primers (**Table S6**). Four primers were designed such that the miRNA and miRNA* sequences are substituted by the desired 21 mers. Since in osa-MIR528 the miRNA is on the forward strand, primer I contains the amiRNA in sense orientation, primer II its reverse complement, primer III the amiRNA* sequence in sense and primer IV the amiRNA* sequence in antisense orientation. The plasmid information for pNW55 has been integrated into the online WMD2 platform, and all appropriate primer sequences, needed for customization of pNW55, can be retrieved using the primer design function of WMD2. For each miRNA construct three modification PCRs were performed with primers G-4368+II, I+IV and III+G-4369 on pNW55 as template, yielding fragments of 256, 87 and 259 bp lengths, respectively. The three resulting fragments were gel purified (Promega) and then fused by one PCR with the two flanking primers G-4368 and G-4369 on a mixture of 1 µl from each previous PCR as template. The fusion product of 554 bp was again gel purified (Promega), cloned into pGEM®-T Easy Vector (Promega), sequence verified, excised with BamHI/KpnI and transferred into the multiple cloning site of the binary vector IRS154 (a pCambia derivative, provided by Emmanuel Guiderdoni, CIRAD, France). In IRS154, the expression of the transgene is driven by the maize ubiquitin promoter and first intron (GenBank accession number: S94464, basepair-positions 2-1993). All six amiRNA plant expression vectors were transformed into *Agrobacterium tumefaciens* strains *LBA4404* and *EHA105.*

### Rice transformation and culture

Two rice varieties, Nipponbare (japonica) and IR64 (indica) were transformed with the transgenes according to modified protocols from references [44] and [45], respectively, and selected on hygromycin. Control plants were obtained by transformation with the empty binary vector IRS154. All regenerated T_0_ transgenic plants were genotyped for the presence of the transgenic *Hygromycin B Phosphotransferase* gene (*Hpt*) with the endogenous *Gos5* gene (AF093635, Os05g48630) as control; see **Table S4** for primer sequences. Plants were grown in a greenhouse in Los Baños, Philippines under natural photoperiod (December 2006–May 2007) at 29 °C (day) and 23 °C (night).

### Small RNA blots

Total RNA was isolated from rice leaves (*Spl11*, *Pds*) and young panicles (*Eui1/CYP716D1*) using Trizol (Invitrogen). Six μg of total RNA was resolved on a 17% PAGE under denaturing conditions and transferred onto a charged nylon membrane (Nytran SuPer Charge, Whatman® Schleicher & Schuell) by semi-dry blotting [26]. The blot was hybridized with five pmol of a radioactively end-labeled oligo probe complementary to the mature amiRNA in PerfectHyb™ Plus (Sigma-Aldrich) hybridization buffer at 38°C overnight. After hybridization, the blot was briefly rinsed with 2×SSC, 0.2% SDS, washed twice for 20 min with 2×SSC, 0.2% SDS at 42°C and exposed to Kodak BioMax MS film for 4 h at room temperature. The blots shown in **Figure 3** were exposed simultaneously onto the same film.

### RT-PCR/qRT-PCR

Primary transgenic lines for each amiRNA vector were selected based on their phenotype. Total RNA was extracted from leaves at tillering stage (*Pds* and *Spl11* genes) or from flowering panicles at heading stage (*Eui1*/*CYP714D1* gene), using Trizol (Invitrogen). The primary RT-PCR screen was performed with a one-step RT-PCR kit (Qiagen) on 40 ng total RNA, and two transgenic lines for each transgene were further analyzed by quantitative Real Time RT-PCR and compared [46] to an empty vector control plant (**Figure 3**) using the primers G-13120, G-13121 (*Spl11*); G-14494, G-14495 (*Pds*); G-13110, G-13111 (*Eui1/CYP714D1*). Reverse transcription (Invitrogen) was performed with 2 µg of total RNA. PCR amplification was carried out in the presence of the double-strand DNA-specific dye SYBR Green (Invitrogen-Molecular Probes®) and monitored in real time with the Opticon Continuous Fluorescence Detection System (MJR). *GAPDH* (Os04g40950, primers G-12061, G-12062) served as standard. All primers are listed in **Table S4**; more detailed protocols and PCR conditions are available upon request.

### Cleavage site mapping

The amiRNA-guided cleavage sites were mapped by RACE-PCR [26]. From 30 µg total RNA extracted from one plant per transgenic line (Trizol), the mRNA fraction was enriched (Oligotex® mRNA Mini Kit, Qiagen). 5′-RACE reactions (GeneRacer™ Kit, Invitrogen) were performed on 250 ng mRNA following the manufacturers instructions, but omitting the dephosphorylation and de-capping steps. PCRs and nested PCRs were performed with the GeneRacer™ primers (Invitrogen) and the gene specific primers G-15552, G-13121 (*Spl11*), G-15553, G-13117 (*Pds*) and G-15556, G-15554 (*Eui1*/*CYP714D1*); see **Table S4** **.** PCR products were cloned into the pCR®4-TOPO Vector (Invitrogen) and the DNA sequence of the 5′-prime-end was determined by dideoxy sequencing between 21 and 42 clones for each transgenic line.

### Genomic DNA blot

Three transgenic lines for each transgene with reduced expression of the target genes (assessed by RT-PCR) were subjected to DNA blot analysis to determine the copy number of the transgene. Fifteen μg genomic DNA of each sample was digested with BamHI, separated on a 1% agarose gel, and transferred onto a nylon membrane (Millipore) by capillary blotting. Two different DIG-labeled probes (NOS terminator and *Hpt* gene) were produced by PCR labeling (PCR DIG Probe Synthesis Kit, Roche), and the blots were hybridized according to the “DIG Applications Manual for Filter Hybridization” (Roche); see **Table S4** for primer sequences.

### Segregation analysis

Mature seeds from three T_0_ pNW78 Nipponbare transgenic lines (NB_pNW78_02, NB_pNW78_16, NB_pNW78_39, all targeting *Pds*), one Nipponbare empty vector control plant, and wild type Nipponbare were planted. After 3 weeks, the phenotype of the T_1_ seedlings was observed and the segregation ratios recorded. Six T_1_ transgenic plants (three plants with normal phenotype and three albino plants) were analyzed by PCR to confirm that the phenotype co-segregated with the transgene.

### Specificity/Transitivity of the amiRNAs

AmiRNA specificity was assayed on RNA from T_0_ pNW76 IR64 plants. Total RNA from three independent IR64_pNW76 transgenic plants with the expected phenotype and from three IR64 empty vector transgenic plants was extracted from flag-leaf samples at heading stage, using Trizol (Invitrogen). After DNase I (Promega) treatment, RNA concentration was normalized to 30 ng/μl. Transcript abundance for *Spl11* and its homologs **(** **Table S3** **)** was measured synchronously, using the GenomeLab™ GeXP Genetic Analysis System (Beckman-Coulter) with ATPase (Os04g56160) as reference gene following the manufacturer's instructions. After reverse transcription (GenomeLab™ GeXP Start Kit, Beckman-Coulter), a multiplex PCR reaction was conducted with Thermo-Start DNA Polymerase (ABgene). The PCR products were then placed in the GenomeLab™ GeXP Genetic Analysis System for capillary electrophoresis and fragment size analysis. The RT-PCR primers used are listed in **Table S7**.

## Supporting Information

Figure S1 Regenerated NB_pNW78 plant on regeneration medium. A regenerated transgenic Nipponbare plant (NB_pNW78, the transgene targeting *Phytoene Desaturase*) on regeneration medium. Many transgenics with severe albino phenotypes died later upon transfer to soil.
(7.63 MB TIF)

Figure S2 DNA blot analysis of amiRNA transgenes. DNA blot analysis of T_0_ transgenic plants with different probes. (A) Transgenic Nipponbare plants with Nos terminator probe; (B) Transgenic IR64 plants with Nos terminator probe; (C) Transgenic Nipponbare plants with Hpt probe; (D) Transgenic IR64 plants with Hpt probe. “Wt” is wild type Nipponbare as non-transgenic control.
(3.70 MB TIF)

Figure S3 Inheritance of an amiRNA transgene. T_1_ progeny from one NB_pNW78 T_0_ plant that contained one single copy of the transgene. (A) 20-day-old rice seedlings of wild type Nipponbare (Wt) and NB_pNW78 T_1_ transgenic line. (B) 20-day-old rice seedlings; NB_pNW78 transgenic plants show albino leaves and delayed growth; IRS154 is the empty vector control. (C) PCR analysis of NB_pNW78 T_1_ plants. The lower bands are the 127 bp PCR products of Gos5, and the upper bands are the 910 bp PCR products of Hpt primers: (1) 1 kb DNA ladder; (2) IRS154 plasmid; (3) wild type; (4) NB_IRS154 empty vector control plant. (5–7) NB_pNW78 T_1_ plants with normal leaf color; (8–10) NB_pNW78 T_1_ plants with albino phenotype.
(5.71 MB TIF)

Figure S4 Gene expression analysis on *Spl11* and homologs. Gene expression profile of the targeted *Spl11* (Target gene, Os12g38210) and homologs based on multiplex RT-PCR in transgenic IR64_IRS154 (empty vector control, black) and IR64_pNW76 (white) plants relative to ATPase in leaves (GenomeLab™ GeXP Genetic Analysis System, Beckman-Coulter). Homologs and primers are listed in Supplementary Tables S3 and S7. The value of gene expression is given as the mean of three independent transgenic plants (T_0_) and error bars indicate the standard deviation between biological replicates. Gene expression of Os08g37570 and Os06g51130 could not be detected in agreement with the Rice MPSS database (http://mpss.udel.edu/rice/)[1].
* Gene expression difference is significant according to Student's *t* test (P = 0.0002).
1. Nakano, M. et al. Plant MPSS databases: signature-based transcriptional resources for analyses of mRNA and small RNA. *Nucleic Acids Res* 34, D731-735 (2006).
(0.63 MB TIF)

Figure S5 PCR Scheme and Map of pNW55.
(0.69 MB TIF)

Table S1 (0.08 MB DOC)

Table S2 (0.05 MB DOC)

Table S3 (0.08 MB DOC)

Table S4 (0.09 MB DOC)

Table S5 (0.05 MB DOC)

Table S6 (0.07 MB DOC)

Table S7 (0.09 MB DOC)
