# Supplementary figures and images for: Highly Specific Gene Silencing by Artificial miRNAs in Rice
Source: PLoS One. 2008 Mar 19;3(3):e1829. doi: 10.1371/journal.pone.0001829 (PMC2262943; doi:10.1371/journal.pone.0001829)

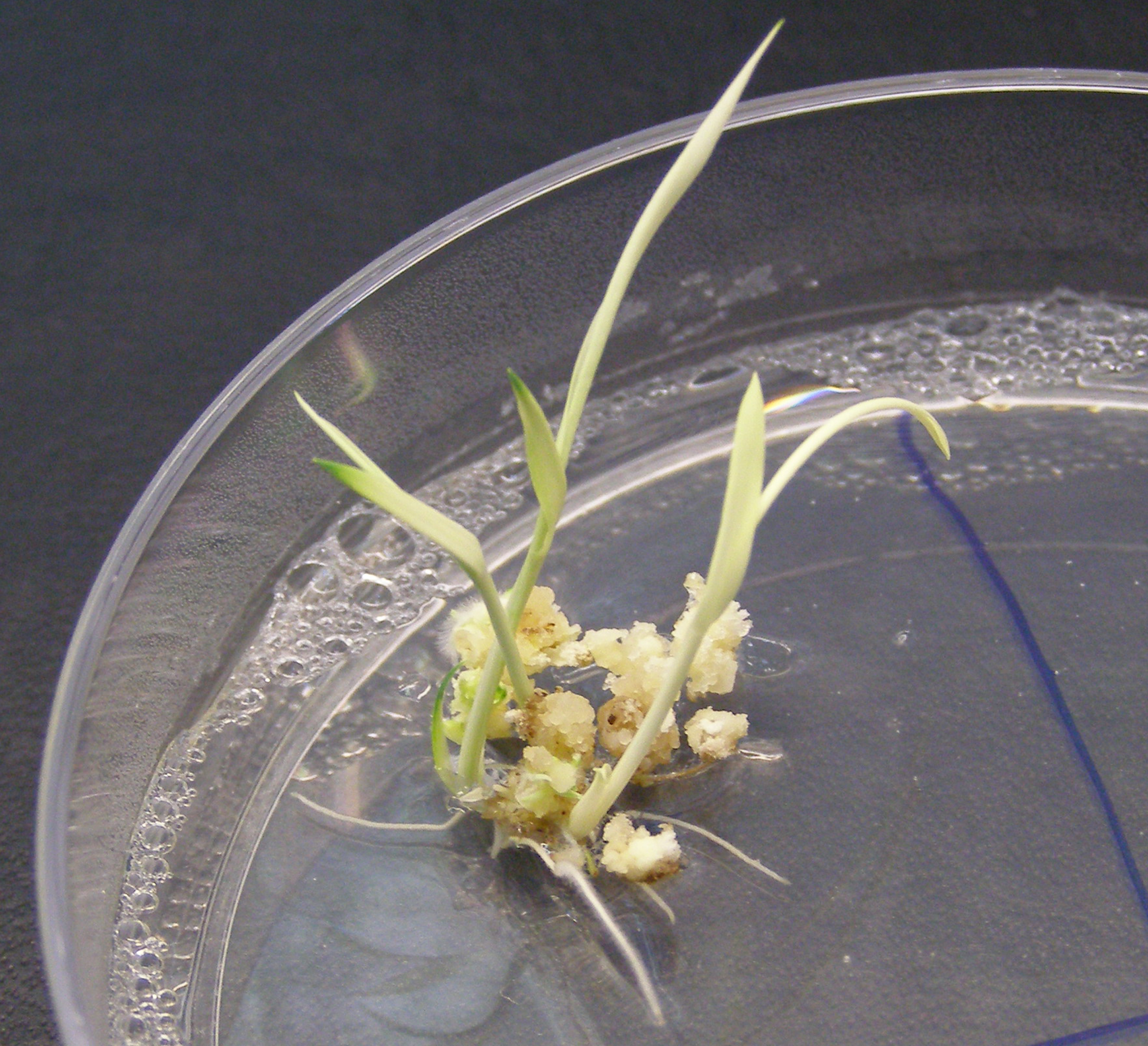

Supplement: Figure S1 — Regenerated NB_pNW78 plant on regeneration medium. A regenerated transgenic Nipponbare plant (NB_pNW78, the transgene targeting Phytoene Desaturase) on regeneration medium. Many transgenics with severe albino phenotypes died later upon transfer to soil. (7.63 MB TIF) [file pone.0001829.s001.tif]

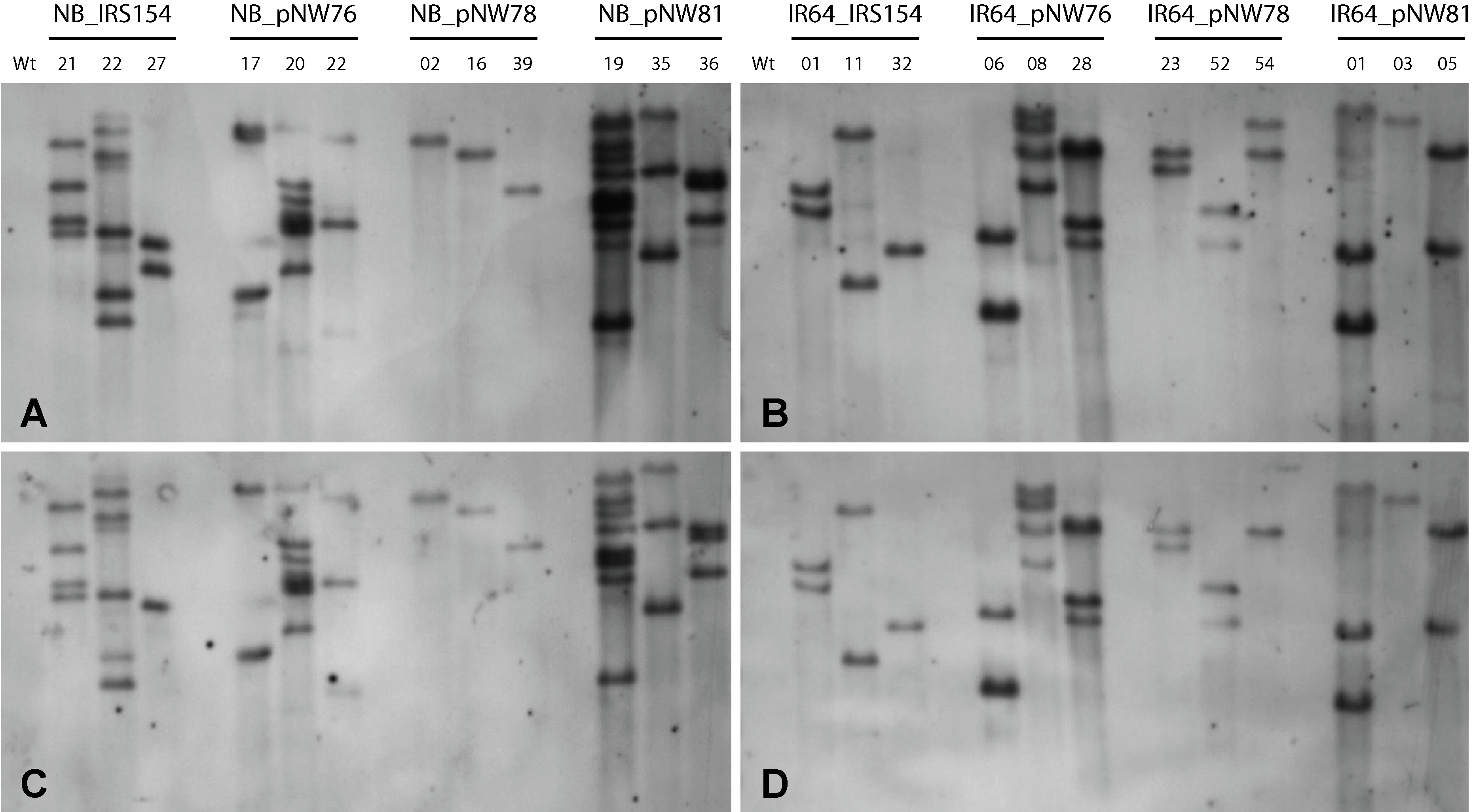

Supplement: Figure S2 — DNA blot analysis of amiRNA transgenes. DNA blot analysis of T0 transgenic plants with different probes. (A) Transgenic Nipponbare plants with Nos terminator probe; (B) Transgenic IR64 plants with Nos terminator probe; (C) Transgenic Nipponbare plants with Hpt probe; (D) Transgenic IR64 plants with Hpt probe. “Wt” is wild type Nipponbare as non-transgenic control. (3.70 MB TIF) [file pone.0001829.s002.tif]

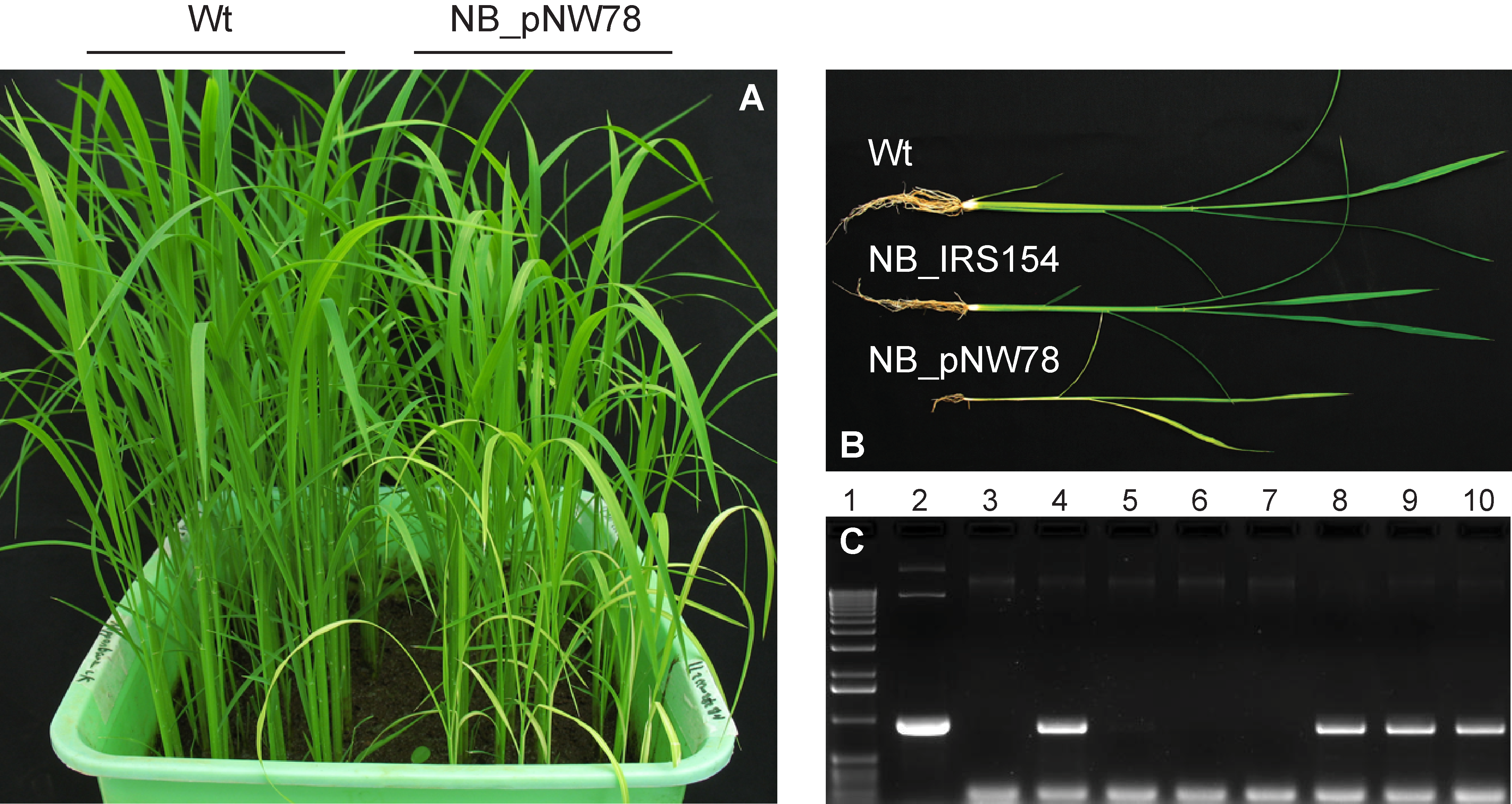

Supplement: Figure S3 — Inheritance of an amiRNA transgene. T1 progeny from one NB_pNW78 T0 plant that contained one single copy of the transgene. (A) 20-day-old rice seedlings of wild type Nipponbare (Wt) and NB_pNW78 T1 transgenic line. (B) 20-day-old rice seedlings; NB_pNW78 transgenic plants show albino leaves and delayed growth; IRS154 is the empty vector control. (C) PCR analysis of NB_pNW78 T1 plants. The lower bands are the 127 bp PCR products of Gos5, and the upper bands are the 910 bp PCR products of Hpt primers: (1) 1 kb DNA ladder; (2) IRS154 plasmid; (3) wild type; (4) NB_IRS154 empty vector control plant. (5–7) NB_pNW78 T1 plants with normal leaf color; (8–10) NB_pNW78 T1 plants with albino phenotype. (5.71 MB TIF) [file pone.0001829.s003.tif]

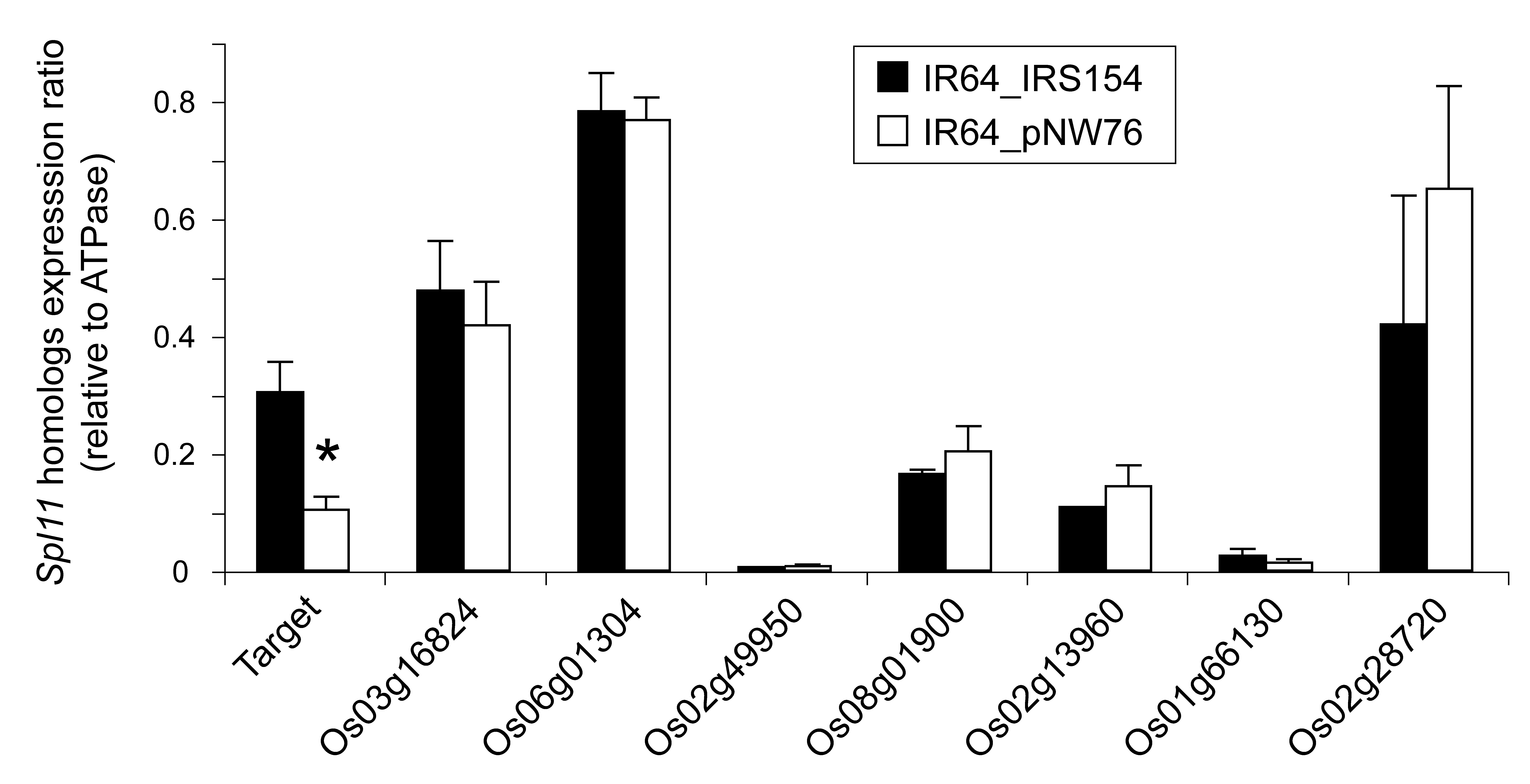

Supplement: Figure S4 — Gene expression analysis on Spl11 and homologs. Gene expression profile of the targeted Spl11 (Target gene, Os12g38210) and homologs based on multiplex RT-PCR in transgenic IR64_IRS154 (empty vector control, black) and IR64_pNW76 (white) plants relative to ATPase in leaves (GenomeLab™ GeXP Genetic Analysis System, Beckman-Coulter). Homologs and primers are listed in Supplementary Tables S3 and S7. The value of gene expression is given as the mean of three independent transgenic plants (T0) and error bars indicate the standard deviation between biological replicates. Gene expression of Os08g37570 and Os06g51130 could not be detected in agreement with the Rice MPSS database (http://mpss.udel.edu/rice/)[1]. * Gene expression difference is significant according to Student's t test (P = 0.0002). 1. Nakano, M. et al. Plant MPSS databases: signature-based transcriptional resources for analyses of mRNA and small RNA. Nucleic Acids Res 34, D731-735 (2006). (0.63 MB TIF) [file pone.0001829.s004.tif]

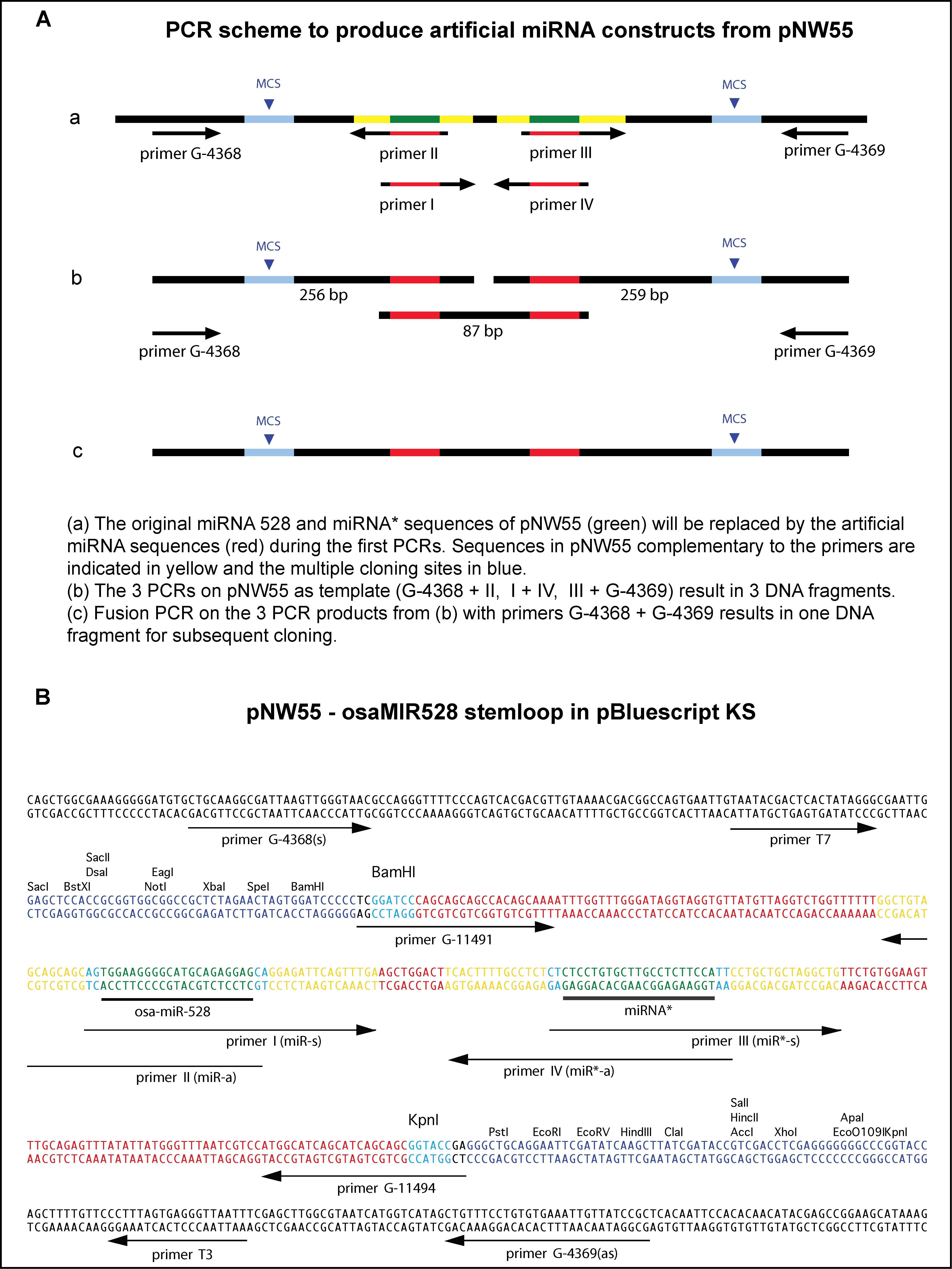

Supplement: Figure S5 — PCR Scheme and Map of pNW55. (0.69 MB TIF) [file pone.0001829.s005.tif]
