# Supplementary material for: Highly Specific Gene Silencing by Artificial miRNAs in Rice
Source: PLoS One. 2008 Mar 19;3(3):e1829. doi: 10.1371/journal.pone.0001829 (PMC2262943; doi:10.1371/journal.pone.0001829)
Supplement: Table S1 — (0.08 MB DOC) [file pone.0001829.s006.doc]

**Table S1** Total plant heights^a^, uppermost internodes lengths^a^ and panicle exsertion^a^ of T_0_ transgenic pNW81 Nipponbare and IR64 plants^b^.

**Nipponbare:**

|  | **Nipponbare** | | |
| --- | --- | --- | --- |
|  | **IRS154** (n=9) | **pNW81** (n=9) | Welch Two Sample **t-test** ^c^ |
| Total plant height (cm) | 66.5 ± 1.82 | 70.4 ± 6.55 | t = -1.7407  df = 9.228  p = 0.1149 |
| Uppermost internodes length (cm) | 24.3 ± 1.74 | 27.36 ± 1.20 | t = -4.3043  df = 14.244  p = 0.00070 |
| Panicle exsertion (cm) | 1.44 ± 0.529 | 4.67± 1.34 | t = -6.7367  df = 10.439  p = 4.158e-05 |

**IR64 first transformation and planting:**

|  | **IR64** | | |
| --- | --- | --- | --- |
|  | **IRS154** (n=9) | **pNW81** (n=9) | Welch Two Sample **t-test** ^c^ |
| Total plant height (cm) | 91.9 ± 1.60 | 101.2 ± 4.73 | t = -5.6078  df = 9.799  p = 0.00024 |
| Uppermost internodes length (cm) | 27.51 ± 0.918 | 33.72 ± 3.04 | t = -5.8528  df = 9.441  p = 0.00020 |
| Panicle exsertion (cm) | 0.31 ± 0.176 | 3.97 ± 0.915 | t = -11.7669  df = 8.594  p = 1.360e-06 |

**IR64 replication** ^b^**:**

|  | **IR64** | | |
| --- | --- | --- | --- |
|  | **IRS154** (n=24) | **pNW81** (n=24) | Welch Two Sample **t-test** ^c^ |
| Total plant height (cm) | 96.1 ± 5.27 | 109.2 ± 8.75 | t = -6.2882  df = 37.715  p = 2.364 e-07 |
| Uppermost internodes length (cm) | 29.19 ± 2.59 | 36.68 ± 3.81 | t = -7.9594  df = 40.508  p = 8.213e-10 |
| Panicle exsertion (cm) | 1.34 ± 1.01 | 3.83 ± 3.37 | t = -3.4711  df = 27.12  p = 0.00175 |

^a^ Data are presented as the mean ± standard deviation of the respective T_0_ transgenic plants. All transgenic lines that showed a phenotype were compared to the same number (n) of empty-vector control plants (IRS154), taking the tallest plants.

^b^ The comparison of IR64_pNW81, and its corresponding IR64_IRS154 was replicated (rice transformation and planting) in a different season (Philippines).

^c^ T-tests were performed using the statistical software package “R” ([http://www.r-project.org](http://www.r-project.org/)).
