# Supplementary material for: Highly Specific Gene Silencing by Artificial miRNAs in Rice
Source: PLoS One. 2008 Mar 19;3(3):e1829. doi: 10.1371/journal.pone.0001829 (PMC2262943; doi:10.1371/journal.pone.0001829)
Supplement: Table S2 — (0.05 MB DOC) [file pone.0001829.s007.doc]

**Table S2** Segregation analysis of the phenotype in progeny (T_1_ generation) of 3 amiRNA transgenic lines (NB_pNW78).

| Line | Copy number of transgene | Number of seeds germinated | Albino seedling phenotype | Normal seedlings | χ^2^ (3:1)^a^ |
| --- | --- | --- | --- | --- | --- |
| Nipponbare wild type | - | 28 | 0 | 28 | - |
| NB_IRS154_21 | 4 | 30 | 0 | 30 | - |
| NB_pNW78_02 | 1 | 30 | 22 | 8 | 0.044 |
| NB_pNW78_16 | 1 | 22 | 15 | 7 | 0.546 |
| NB_pNW78_39 | 1 | 37 | 28 | 9 | 0.009 |

^a^ Limit is 3.84 for α = 0.05, one degree of freedom.
