# Supplementary material for: Highly Specific Gene Silencing by Artificial miRNAs in Rice
Source: PLoS One. 2008 Mar 19;3(3):e1829. doi: 10.1371/journal.pone.0001829 (PMC2262943; doi:10.1371/journal.pone.0001829)
Supplement: Table S3 — (0.08 MB DOC) [file pone.0001829.s008.doc]

**Table S3** Gene list of *Spl11* (Os12g38210) and its 9 closest homologs in the rice genome.

| **TIGR locus identifier** | **Annotation in TIGR5** | **Blast**  **score** | **Blast**  **e-value** | **Identity^a^** |
| --- | --- | --- | --- | --- |
| LOC_Os12g38210.1^b^ | spotted leaf protein 11,  putative, expressed | 8640 | 0 | 2517/2517 (100.0%) |
| LOC_Os12g38210.2^b^ | spotted leaf protein 11,  putative, expressed | 8224 | 0 | 2490/2517 (98.9%) |
| LOC_Os03g16824.1 | spotted leaf protein 11,  putative, expressed | 2493 | 2.2e-112 | 1707/3199 (53.4%) |
| LOC_Os06g01304.1^c^ | spotted leaf protein 11,  putative, expressed | 1937 | 8.4e-96 | 1621/3383 (47.9%) |
| LOC_Os06g01304.2^c^ | spotted leaf protein 11,  putative, expressed | 1937 | 8.4e-96 | 1424/2817 (50.6%) |
| LOC_Os02g49950.1 | spotted leaf protein 11,  putative, expressed | 1586 | 4.3e-67 | 1447/2990 (48.4%) |
| LOC_Os08g37570.1 | spotted leaf protein 11,  putative, expressed | 1323 | 7.8e-55 | 1403/2971 (47.2%) |
| LOC_Os08g01900.1 | ubiquitin-protein ligase,  putative, expressed | 898 | 4.3e-42 | 1548/3463 (44.7%) |
| LOC_Os02g13960.1 | spotted leaf protein 11,  putative, expressed | 647 | 2.7e-31 | 1361/2971 (45.8%) |
| LOC_Os01g66130.1 | armadillo-repeat containing  protein, putative, expressed | 797 | 2.4e-27 | 1434/3430 (41.8%) |
| LOC_Os06g51130.1 | spotted leaf protein 11,  putative, expressed | 749 | 2.8e-25 | 1344/2872 (46.8%) |
| LOC_Os02g28720.1 | spotted leaf protein 11,  putative, expressed | 460 | 6.4e-12 | 1218/2811 (43.3%) |

^a^ Identity of the CDS DNA sequences to Os12g38210.1 was determined by a pair wise Needleman-Wunsch alignment using the EMBOSS [1] program ‘needle’ with default parameters.

^b^ amiRNA target gene. There are two splice variants with slightly different CDS sequences (30 bp indel).

^c^ Two splice variants.

1. Rice P, Longden I, Bleasby A (2000) EMBOSS: the European Molecular Biology Open Software Suite. Trends Genet 16: 276-277.
