# Supplementary material for: Highly Specific Gene Silencing by Artificial miRNAs in Rice
Source: PLoS One. 2008 Mar 19;3(3):e1829. doi: 10.1371/journal.pone.0001829 (PMC2262943; doi:10.1371/journal.pone.0001829)
Supplement: Table S4 — (0.09 MB DOC) [file pone.0001829.s009.doc]

**Table S4** Additional primers^a^.

| **RT-PCR:** | | | | |
| --- | --- | --- | --- | --- |
| *GAPDH*  (Os04g40950) | G-12061 | Forward | CTGCAACTCAGAAGACCGTTG | 329 bp |
|  | G-12062 | Reverse | CCTGTTGTCACCCTGGAAGTC |  |
| *Spl11*  (Os12g38210) | G-13120 | Forward | CGAGCGGATGAGCAGATTC | 231 bp |
|  | G-13121 | Reverse | AAGTGACCAGGATGCTACTCAATC |  |
| *Pds*  (Os03g08570) | G-14494 | Forward | CATTGCCTGCACCCTTAAAT | 390 bp |
|  | G-14495 | Reverse | GAACCTCACCACCCAAAGAG |  |
| *Eui1*  (Os05g40384) | G-13110 | Forward | GGTTCGAGTTCACGCTCTC | 243 bp |
|  | G-13111 | Reverse | AATGGTAATCCTCTGTTGTCTCTG |  |
| **Amplification of Osa-MIR528 from genomic DNA:** | | | | |
| Osa-MIR528 | G-11491 | Forward | tcggatccCAGCAGCAGCCACAGCAAA | 261 bp |
|  | G-11494 | Reverse | tcggtaccGCTGCTGATGCTGATGCCAT |  |
| **Southern blot and genotyping:** | | | | |
| *Hpt* |  | Forward | AGAATCTCGTGCTTTCAGCTTCGA | 910 bp |
|  |  | Reverse | TCAAGACCAATGCGGAGCATATAC |  |
| Nos terminator |  | Forward | TTATCCTAGTTTGCGCGCTA | 180 bp |
|  |  | Reverse | GAATCCTGTTGCCGGTCTTG |  |
| *Gos5* (AF093635, Os05g48630) |  | Forward | TACGGCGAGAAGAGCGTCTACT | 127 bp |
|  |  | Reverse | CGAACGTCTCGAAGAACTTGCT |  |
| **Cleavage site mapping^b^:** | | | | |
| *Spl11* | G-15552 | 1st PCR | ACATCCTGGGCTGCCTAAACAAATTCTCA | 259 bp |
|  | G-13121 | Nested PCR | AAGTGACCAGGATGCTACTCAATC | 116 bp |
| *Pds* | G-15553 | 1st PCR | AGGAACTCCCACCAACTTCTCCAGCTTCTT | 263 bp |
|  | G-13117 | Nested PCR | CATCTCCAGTTATTTGAGTTCCATC | 145 bp |
| *Eui1* | G-15556 | 1st PCR | GCTGGTAAAATGGTAATCCTCTGTTGTCTCTG | 146 bp |
|  | G-15554 | Nested PCR | GTAAAATGGTAATCCTCTGTTGTCTCTGTATGTGT | 129 bp |

**^a^**Sequence not matching the template is depicted in lower case.

^b^Given are the gene-specific reverse primers used in combination with the GeneRacer^TM^ 5’ Primer and GeneRacer^TM^ 5’ Nested Primer (Invitrogen).
