# Supplementary material for: Highly Specific Gene Silencing by Artificial miRNAs in Rice
Source: PLoS One. 2008 Mar 19;3(3):e1829. doi: 10.1371/journal.pone.0001829 (PMC2262943; doi:10.1371/journal.pone.0001829)
Supplement: Table S5 — (0.05 MB DOC) [file pone.0001829.s010.doc]

**Table S5** PCR protocols for directed mutagenesis of pNW55^a^.

| **Modification PCRs on template clone pNW55:** | | |
| --- | --- | --- |
| Primer | Product size | PCR protocol |
| G-4368 + primer II | 256 bp | 95°C 2 min;  34 cycles of 95°C 30s, 55°C 30s, 72°C 30s; 72°C 7 min |
| Primer I + primer IV | 87 bp |
| Primer III + G-4369 | 259 bp |
| **Fusion PCR on a mixture of all PCR products of the Modification PCRs:** | | |
| Primer | Product size | PCR protocol |
| G-4368 + G-4369 | 554 bp | 95°C 2 min;  34 cycles of 95°C 30s, 55°C 30s, 72°C 1min; 72°C 7 min |

^a^ The corresponding PCR schema is depicted in **Figure S5**. Protocols and PCR schema were adapted from Schwab, et al. 2006 [1].

1. Schwab R, Ossowski S, Riester M, Warthmann N, Weigel D (2006) Highly specific gene silencing by artificial microRNAs in Arabidopsis. Plant Cell 18: 1121-1133.
