# Supplementary material for: Highly Specific Gene Silencing by Artificial miRNAs in Rice
Source: PLoS One. 2008 Mar 19;3(3):e1829. doi: 10.1371/journal.pone.0001829 (PMC2262943; doi:10.1371/journal.pone.0001829)
Supplement: Table S6 — (0.07 MB DOC) [file pone.0001829.s011.doc]

**Table S6** Primer sequences for directed mutagenesis of pNW55.

| **Clone** | **Primer** | **Sequence^a^** | **Alias** | **Comment** |
| --- | --- | --- | --- | --- |
| all | Universal | CTGCAAGGCGATTAAGTTGGGTAAC | G-4368 | Forward |
|  | Universal | GCGGATAACAATTTCACACAGGAAACAG | G-4369 | Reverse |
| pNW75 | primer I | AGtaaggcgagtgattcatgcgtCAGGAGATTCAGTTTGA | G-11669 | miR-s |
|  | primer II | TGacgcatgaatcactcgccttaCTGCTGCTGCTACAGCC | G-11670 | miR-a |
|  | primer III | CTacgcaagaaacactcgccttaTTCCTGCTGCTAGGCTG | G-11671 | miR*-s |
|  | primer IV | AAtaaggcgagtgtttcttgcgtAGAGAGGCAAAAGTGAA | G-11672 | miR*-a |
| pNW76 | primer I | AGtatagggtattgatacgctggCAGGAGATTCAGTTTGA | G-11673 | miR-s |
|  | primer II | TGccagcgtatcaataccctataCTGCTGCTGCTACAGCC | G-11674 | miR-a |
|  | primer III | CTccagcctatgaataccctataTTCCTGCTGCTAGGCTG | G-11675 | miR*-s |
|  | primer IV | AAtatagggtattcataggctggAGAGAGGCAAAAGTGAA | G-11676 | miR*-a |
| pNW77 | primer I | AGttaagaattactatgcaggccCAGGAGATTCAGTTTGA | G-11677 | miR-s |
|  | primer II | TGggcctgcatagtaattcttaaCTGCTGCTGCTACAGCC | G-11678 | miR-a |
|  | primer III | CTggcctccattgtaattcttaaTTCCTGCTGCTAGGCTG | G-11679 | miR*-s |
|  | primer IV | AAttaagaattacaatggaggccAGAGAGGCAAAAGTGAA | G-11680 | miR*-a |
| pNW78 | primer I | AGtaaagagcgaacatggtcgacCAGGAGATTCAGTTTGA | G-11681 | miR-s |
|  | primer II | TGgtcgaccatgttcgctctttaCTGCTGCTGCTACAGCC | G-11682 | miR-a |
|  | primer III | CTgtcgagcatcttcgctctttaTTCCTGCTGCTAGGCTG | G-11683 | miR*-s |
|  | primer IV | AAtaaagagcgaagatgctcgacAGAGAGGCAAAAGTGAA | G-11684 | miR*-a |
| pNW81 | primer I | AGttgagaactatgcacgggcgcCAGGAGATTCAGTTTGA | G-11693 | miR-s |
|  | primer II | TGgcgcccgtgcatagttctcaaCTGCTGCTGCTACAGCC | G-11694 | miR-a |
|  | primer III | CTgcgccggtggatagttctcaaTTCCTGCTGCTAGGCTG | G-11695 | miR*-s |
|  | primer IV | AAttgagaactatccaccggcgcAGAGAGGCAAAAGTGAA | G-11696 | miR*-a |
| pNW82 | primer I | AGtagttcacgacttactaggtgCAGGAGATTCAGTTTGA | G-11697 | miR-s |
|  | primer II | TGcacctagtaagtcgtgaactaCTGCTGCTGCTACAGCC | G-11698 | miR-a |
|  | primer III | CTcaccttgtatgtcgtgaactaTTCCTGCTGCTAGGCTG | G-11699 | miR*-s |
|  | primer IV | AAtagttcacgacatacaaggtgAGAGAGGCAAAAGTGAA | G-11700 | miR*-a |

^a^Artificial miRNA and amiRNA* sequences to be replaced (and thus not matching the template) are in lower case. For the PCR protocol see **Table S5.**
