# Supplementary material for: Highly Specific Gene Silencing by Artificial miRNAs in Rice
Source: PLoS One. 2008 Mar 19;3(3):e1829. doi: 10.1371/journal.pone.0001829 (PMC2262943; doi:10.1371/journal.pone.0001829)
Supplement: Table S7 — (0.09 MB DOC) [file pone.0001829.s012.doc]

**Table S7** Primer sequences used for RT-PCR on *Spl11* homologs for the GenomeLab™ GeXP Genetic Analysis System (Beckman-Coulter).

| **TIGR locus identifier** | **Orientation** | **Primer sequences**  (with universal sequence in lower case) | **Product size** |
| --- | --- | --- | --- |
| LOC_Os12g38210 | FWD | aggtgacactatagaataCGGCTGTCATGCTACATCTG | 167 bp |
|  | REV | gtacgactcactatagggaGCCTTCCTCTTCCCCCTATC |  |
| LOC_Os01g66130 | FWD | aggtgacactatagaataAGGTTTTGCACCCTGGTTCT | 183 bp |
|  | REV | gtacgactcactatagggaTACCCTCTCCCAACCCTGAC |  |
| LOC_Os02g13960 | FWD | aggtgacactatagaataTTGCTCCGCTACTGCCTATT | 188 bp |
|  | REV | gtacgactcactatagggaTTGGGCATGAGAACTCCTTT |  |
| LOC_Os02g49950 | FWD | aggtgacactatagaataCGGAATCTGGTGCAATATCC | 207 bp |
|  | REV | gtacgactcactatagggaCCCCTTCCTCAACACCTGTA |  |
| LOC_Os02g28720 | FWD | aggtgacactatagaataGGATGACCAATGCAAACGTA | 210 bp |
|  | REV | gtacgactcactatagggaGGATACCAGCAGCCTTCCTC |  |
| LOC_Os03g16824 | FWD | aggtgacactatagaataGGATCAGCAAGGAACAAGGA | 215 bp |
|  | REV | gtacgactcactatagggaTTCGCTCAAGAAGCTGGATT |  |
| LOC_Os06g01304 | FWD | aggtgacactatagaataATATGAGCGATCCTGCATCC | 230 bp |
|  | REV | gtacgactcactatagggaTTTTGCCGCCTTCTTATCAC |  |
| LOC_Os06g51130 | FWD | aggtgacactatagaataATGCTCAACAATGGCAACAA | 239 bp |
|  | REV | gtacgactcactatagggaTCCCTTCCTTGATGATCTGC |  |
| LOC_Os08g01900 | FWD | aggtgacactatagaataTTGGCAAACCTTGCTACCAT | 255 bp |
|  | REV | gtacgactcactatagggaGTGCCTGACTGTGACAATGC |  |
| LOC_Os08g37570 | FWD | aggtgacactatagaataGAAGTCAAGGAGCAGGTGGA | 266 bp |
|  | REV | gtacgactcactatagggaCGCTGATGAGCTTCTTGATG |  |
| LOC_Os04g56160 (ATPase) | FWD | aggtgacactatagaataACGCTTCACTTGAGGCACTT | 307 bp |
|  | REV | gtacgactcactatagggaGGACAGCGCTGATAATGACA |  |
